# The impact of occupation-based problem-solving strategies training in women with breast cancer

**DOI:** 10.1186/s12955-019-1170-5

**Published:** 2019-06-17

**Authors:** Sedef Şahin, Mine Uyanık

**Affiliations:** Faculty of Health Sciences, Department of Occupational Therapy, Hacettepe University, Samanpazarı, 06100 Ankara, Turkey

**Keywords:** Activity performance, Breast Cancer, Occupational therapy, Problem solving, Rehabilitation, Quality of life

## Abstract

**Background:**

By identifying the occupations of women with breast cancer who have performance problems, to examine the impact of the application of occupation-based problem-solving strategies (OB-PSS) training on cancer-related fatigue, depression, and quality of life.

**Methods:**

The study comprises 22 women outpatients in the clinic. Socio- demographic and Clinical Features Information Collection Form, Canadian Occupational Performance Measure (COPM), Cancer Fatigue Scale (CFS), Beck Depression Inventory (BDI), The European Organization for Research and Treatment of Cancer Core Quality of Life Questionnaire C-30 and BR23 (EORTC QOL-C30 - EORT QOL-BR23) tests have been applied to survivors. OB-PSS training was conducted on a face-to-face basis once a week for 6 weeks.

**Results:**

When activity distribution results in accordance with the performance areas are studied, women with breast cancer were seen to suffer problems mostly in their most productive areas (housework management). As a means of solving these performance problems, they developed adaptive strategies like including additional new steps to these activities. Statistically meaningful results have been obtained between measurements before and after the treatment process through all tests (*p* < 0.01).

**Conclusions:**

OB-PSS provides positive gains in women with breast cancer in terms of a reduction in the degree of cancer-related fatigue and depression, and a progress in performance and satisfaction levels particularly in activities where performance problems are experienced and an improvement in quality of life. OB-PSS training could be used as an appropriate rehabilitation approach for coping with problems in women’ life with breast cancer.

## Background

Cancer is a serious and chronic disease, as well as being a disease-bearing uncertainty, bringing to one’s mind death in great suffering and pains and leading to feelings of panic and anxiety [1] . Breast cancer is the type of cancer which is most prevalent in women in the world [2]. Life expectancy in survivors with breast cancer has been increased thanks to early diagnosis and more effective treatment of cancer with the help of current developments. The increase in the number of surviving breast cancer patients after treatment leads to an increase in secondary problems accompanied by the disease and its treatment [3]. Among those are both physical and psychological symptoms such as pain, fatigue, exhaustion, anxiety, limitation in daily routines and lack of participation [4, 5]. It is known that people’s life quality is adversely affected in the case of one or more of these symptoms [6].

Considering the increase in the number of cancer patients in recent years, there is a growing need for further research into the determination of problems people experience during their daily routines and their intervention methods. As each and every disease and its symptoms will tend to vary from one person to another, the severity of these symptoms and the degree of capability to cope with them will also be different. Involving individual-oriented and holistic approaches, occupational therapy (OT) is a rehabilitation area which has a growing interest within studies in this field [7–9]. Considering the studies carried out into the effectiveness of OT interventions, it has been observed that procedures like problem solving strategy (PSS), which are conducted towards activities in which survivors experience performance problems, positively influence individual coping skills [8–10]. The importance of effectiveness of individual coping skills in increasing one’s activity and role performance is also an issue that has been strongly emphasized in recent years [9, 10].

PSS was originally developed as a therapy for depression and adapted later for use in medical settings [11, 12]. It has been used to help cancer patients develop and evaluate various solutions to the difficulties they encounter in life [10]. PSS consists of various steps such as detecting the problem, choosing a meaningful, purposeful and observable target, brainstorming possible solutions, identifying the advantages and disadvantages of each and every possible solution, deciding on the solution, implementation and assessment in accordance with the feedback obtained [10, 11]. In OB-PSS, the problem is identified using COPM, which is one of the holistic approaches in the field of OT, from amongst all the activities in the fields of self care, productivity and free time performance and all other PSS steps are monitored. The most important component of PSS is individual activation, in which the therapist does not offer specific solutions. Thus, the individual becomes the active director of recovery.

The objective of our study, which is based upon the hypothesis that there is a lack of adequate research into the influences of activity-based applications on cancer patients, is to establish the fact that women with breast cancer experience performance problems, and to study the influence of OB-PSS on activity performance and satisfaction, cancer-based fatigue, depression and quality of life.

## Methods

### Selection and description of participants

The study was conducted at the outpatient Oncology Clinic of the University Hospital, by convenience sampling from May 2016 to May 2017. Criteria to get involved in the study: (1) being women between 18 and 64 years of age, (2) diagnosed as stage 1 or 2 breast cancer stage, (3) having received chemotherapy in the last 6 months, (4) being cooperative. Non-participation criteria: (1) having been diagnosed with breast cancer at stages 3–4, (2) having received radiotherapy in the last 6 months, (3) having another secondary chronic disease, (4) having received hormone therapy in the last 9 months, (5) being a part of any other rehabilitation program.

During the study period, the patients who applied to the clinics were screened as potential participants. 25 women were eligible and of these, three women subsequently met exclusion criteria: had started to receive the radiotherapy at the same time during the study. Therefore, total 22 women who applied to the as outpatients with breast cancer diagnosis were included in the study (the sample number has turned out to be 22, taking the error margin as 5% (*p* = 0.05) at an 80% strength through statistical power analysis).

Women with participant criteria were given the rating scales that are described in detail below. OB-PSS was conducted one session (60–90 min) per week for 6 weeks. Participants were assessed again with the same scales after the intervention and the results were compared.

### Measurements

#### Socio-demographic and clinical features form

So as to comprehensively evaluate women with breast cancer, with a compilation from other similar studies, an information collection form was specifically designed and used to examine socio-demographic features such as age, gender, duration of disease (months), number of cures, cancer stage, medication, marital status, educational status, occupation, present condition in terms of employment.

### Primary outcomes

#### Canadian occupational performance measure

COPM, a standard measurement tool was used to identify the occupational performance problems of survivors with breast cancer and measure their sense of satisfaction [13]. The COPM is a client-centered measure that examines self-perceived changes through semi-structured interview in the occupational performance according to three occupational performance areas (self-care, productivity, and leisure) [14]. It has been translated into more than 20 languages in over 35 countries and is commonly used in occupational therapy departments. The test-retest reliability of the COPM is within the acceptable range; intra-class correlation coefficients for patients with chronic diseases and obtained coefficients ranging from 0.73 to 0.93 [15–17].

In COPM, individuals are firstly asked to identify the problematic activities regarding self-care, productivity and leisure, which they normally do, want to do or are restrained from doing in their daily routines. At the second stage, they are asked to give points between 1 and 10 on the likert scale for each of the activities they have identified (1-Not important, 10-Very important). At the third stage, everyone was asked to choose at least 1 and at the most 5 most important activities and then give a point for each activity between 1 and 10 on the likert scale for performance and satisfaction. Performance and satisfaction scores are determined by dividing the sum of performance and satisfaction scores to the number of activities the individual considers important [18, 19].

### Secondary outcomes

#### Cancer fatigue scale

CFS was used to evaluate the fatigue level of survivors with breast cancer. This scale was developed in Japan by Okuyama et al. in 2000. It examines fatigue physically, emotionally and cognitively from a tri-dimensional perspective. CFS was proved to be a valid and reliable tool used to evaluate fatigue both in survivors with breast cancer and in other various types of cancer patients [20–24]. The items in this scale which consists of 15 items is scored between 1 (never) and 5 (always). The score interval for physical function sub-heading is between 0 and 28, for emotional function sub-heading 0–16 and for cognitive function sub-heading between 0 and 16. Maximum total score is 60 points. A high score is the indication of a higher cancer-related fatigue level [20].

#### Beck depression inventory

BDI was used to determine depression levels of survivors with breast cancer. This scale was developed by Beck et al. in 1961 [25]. It evaluates the physical, emotional, cognitive and motivational symptoms observed during depression. Each item is scored between 0 and 3. The points given for each item are added up to calculate the depression score. A high score means that the severity of depression is high [25].

#### The European Organization for Research and Treatment of Cancer quality of life

In our study, EORTC-QOL C30 and BR-23 life quality assessment tests, which are recommended by World Health Organization to be used in the assessment of life quality of cancer patients, and their various versions designed for specific cancer types have been used [26]. EORTC-QOL C-30 test is made up of 30 items under three sub-headings, which are general well-being, functional difficulties and symptom control. First 28 items are scored between 1 (none) and 4 (high), and the last two items between 1 (too bad) and 7 (perfect) on the likert scale. Total score may range between 0 and 100. Items 29 and 30 are related with general well-being. High scores in this section indicate a high life quality, whereas low ones refer to a low life quality. Lower scores in the functional and symptoms domains point to high life quality, while high points come to mean low life quality [27].

EORTC QOL BR-23 is a test made up of 23 items each of which is scored between 1 (none) and 4 (high) on the likert scale, used to assess the quality of life of survivors with breast cancer under two headings, functional and symptoms [28]. Low scores mean high life quality, while high scores denote to low life quality [29].

### Intervention

PSS is a method in which development of skills and strategies in cancer patients are encouraged and certain adaptations are made in line with personal needs [10]. OB-PSS was given to the participants by the authors. The authors and the participants identified problematic activity together with COPM. The authors (therapists) provided guidance by describing the steps involved in the OB-PSS. The authors and the participants found the solution to the problem in this method together again. Later, the participants experienced of the solution way in their real environment on routine life. The authors received feedback from the participants about their experiences. As a result of this interactive method, the professionals give the individual a chance to choose and try to solve the problem-solving strategy in their activity practice. OB-PSS training was conducted in 4 stages totally in 6 sessions.

- Stage 1: Setting a measurable, realistic and attainable goal for the solution of activities that involve performance problems
- Stage 2: Considering and studying the pros and cons of possible solutions by making a brainstorming through the Canadian Model of Occupational Performance
- Stage 3: After deciding on a possible solution, making up a plan and stepping into action
  - *Adaptation of the activity,* making alterations to one or more of the following: who (involving another person), where (making a change in the place), when (changing the time), how (altering the way of application) and what (adding up new steps at the beginning or end of the activity).
  - *Finding out the new activity,*
  - *Planning the steps of the activity* (in accordance with priority),
  - *Bringing together activity-related information and resources*
- Stage 4: Revising the problem-solving process which has been activated with the OB-PSS training, receiving feedback about individual’s experiences and making alterations to the course of action when necessary.

### Statistical method

Data were analyzed using IBM the Statistical Package for the Social Sciences (SPSS) version 23.0 software. The variables were investigated using visual (plots/histograms) and analytical methods (Kolmogorov-Smirnov Test) to determine whether or not they are normally distributed (normal = *P* > .05, not normal = *P* < .05). Paired student-t and Wilcoxon signed rank test were used to test the mean differences between at the beginning and at the end of the intervention. Significance was set an alpha level of 0.05. Quantitative data were described with mean ± standard deviation (X ± SD), qualitative data were described with percent (%) values.

In the analysis of qualitative data of activities, MAXODA 11.0 version was used and content analysis was made [30]. The approach uses a systematic and reproducible process to encode the manifest content of a text, followed by the number of data added to each code [31].

## Results

Twenty two women with breast cancer between ages 46.77 ± 7.99 years participated in this study. Their average duration of disease is found to be 5.77 ± 2.52 (2–12) months and average total number cures taken 5.68 ± 3.21 (2–16) months. Other demographic data are illustrated in Table 1.

**Table 1:** Findings related to the demographic information about women (*N* = 22)

	N	%
Stage of Cancer		
Stage 1	9	40,9
Stage 2	15	59,1
Drug Use		
Yes	19	86.4
No	3	13,6
Drug use for depression		
Yes	8	36,6
No	14	63,4
Marital status		
Married	16	72,7
Single	2	9,1
Widow	4	18,2
Level of education		
Primary school	0	0
Middle school	2	9,1
High school	9	40,9
University	11	50,0
Work status (Before medical treatment)		
Yes	12	54,5
No	10	45,5
Work status (After medical treatment)		
Yes	3	13,6
No	19	86,4

When all the activities determined in accordance with COPM are studied with reference to performance domain distribution, women with breast cancer were seen to suffer mostly from productivity problems (45.5%), followed by self-care (40.9%) and leisure (13.6%) problems. Totally 110 different self-care, productivity and leisure activities were defined, all which survivors wanted to perform yet had difficulty to do so, and OB-PSS training was carried out in line with these identified activities. All the activities that were defined are shown in Table 2.

**Table 2:** All the activities with performance problems determined by COPM

	N	%
SELF-CARE	45	40,90
Personal Care	35	31,81
Dressing (upper body)	17	15,45
Dressing bra	8	7,27
Dressing t-shirt	6	5,45
Dressing shirt	3	2,72
Bathing	13	11,81
Hair washing	7	6,36
Back washing	4	3,63
Body washing	2	1,81
Personal hygiene	5	4,54
Hair scanning	4	3,63
Teeth brushing	1	0,9
Functional Mobilite	5	4,54
In-bed mobilizing	5	4,54
Community Management	5	4,54
Driving a car	3	2,72
Shopping	2	1,81
PRODUCTIVITY	50	45,45
Paid/ Unpaid Work	6	5,45
Writing on whiteboard	3	2,72
Using a computer	1	0,9
Writing on paper	2	1,81
Household Management	44	40,0
Cleaning home	28	25,45
Vacuuming the carpet	11	10,0
Hanging curtains	9	8,18
Mopping the floor	3	2,72
Hanging clothes	2	1,81
Dusting	2	1,81
Ironing	1	0,9
Cooking	16	14,54
Stirring	5	4,54
Opening the jar	5	4,54
Carrying teapot	3	2,72
Using a knife	3	2,72
Play/school	0	0
LEISURE	15	13,65
Quiet Recreation (Hobbies etc.)	8	7,29
Knitting	6	5,45
Reading a book	1	0,9
Painting	1	0,9
Active Recreation (Sports etc.)	3	2,72
Dancing (Tango)	1	0,9
Hiking	1	0,9
Fitness	1	0,9
Socialization	4	3,63
Relatives visit	4	3,63
TOTAL	110	100

Note: *COPM* Canadian Occupational Performance Measure

When the results of the OB-PSS steps applied to survivors are examined, performance problems were seen to have been experienced in a total of 110 activities which were identified by COPM. 110 different targets were defined for solutions to the problems that were identified. During the solution process of these problems and evaluations obtained from their feedback, in 12 activities out of all 110, it was seen that survivors failed to come up with any solution to their performance problems in their activities. In the process of establishing the action plan for the solution to performance problems, it was seen that 58.19% of the survivors preferred to make adaptations to the activities by either making them easier or more fun. In all 103 adaptive strategies that were developed as solutions to problems, 33.9% of them were found to make changes to ‘what’ and 28.2 of them to ‘how’ of the activity (Table 3).

**Table 3:** Findings concerning OB-PSS steps (*N* = 110 activities)

	N	%
Stage 1. Setting a measurable, realistic and attainable goal for the solution of activities	110	100
Stage 2. Considering and studying the pros and cons of possible solutions by making a brainstorming through the Canadian Model of Occupational Performance	110	100
Stage 3. After deciding on a possible solution, making up a plan and stepping into action	110	100
-Adaptation of the activity	64	58.1
Who	10	9.7
Where	19	18.5
When	10	9.7
How	29	28.2
What	35	33.9
-Finding out the new activity	8	7.2
-Planning the steps of the activity	28	25.6
-Bringing together activity-related information and resources	10	9.1
Stage 4. Evaluation of the solution process	110	100
Successful	98	89
Unsuccessful	12	11

Note: *OB-PSS* Occupation-based problem solving strategies

When the results of the activity performance and satisfaction are examined after the OB-PSS training, a statistically significant increase was found (*p* < 0.01). The differences between the results of tests – CFS for fatigue levels, BDI for depression levels, EORTC-QOL-C30 and BR-23 for quality of life – before and after intervention were statistically significant (*p* < 0.01) (Table 4).

**Table 4:** Comparison of COPM, CFS, BDI and EORTC-QOL averages according to pre and post interventions

		Pre-Intervention X ± SD	Post-Intervention X ± SD	t	p
COPM	Performance	2,96 ± 0,97	6,81 ± 1,04	−16,96	< 0.01*
	Satisfaction	2,67 ± 1,33	7,03 ± 1,28	−12,56	< 0.01*
CFS	Physical function	15,59 ± 4,11	7,04 ± 3,73	9,45	< 0.01*
	Affective function	11,77 ± 2,32	7,81 ± 1,73	8,06	< 0.01*
	Cognitive function	3,72 ± 1,54	1,59 ± 1,89	4,57	< 0.01*
	Total function	27,13 ± 4,57	20,40 ± 4,45	6,51	< 0.01*
				Z	p
BDI	Total score	18,86 ± 6,54	6,86 ± 3,82	− 4120	< 0.01*
EORTC-QOL-C30	General Health Status	6,59 ± 2,01	11,27 ± 1,63	−4,14	< 0.01*
	Functional Status	34,36 ± 6,48	22,13 ± 3,74	−4,11	< 0.01*
	Symptom Scale	26,40 ± 3,96	17,95 ± 3,53	− 4,10	< 0.01*
	Total Score	67,36 ± 8,63	51,36 ± 5,00	−4,11	< 0.01*
EORTC-QOL-BR23	Functional Status	18,50 ± 2,46	16,22 ± 2,58	−3,26	< 0.01*
	Symptom Scale	36,18 ± 3,34	27,18 ± 4,39	−3,47	< 0.01*
	Total Score	54,68 ± 5,02	43,40 ± 4,83	−3,57	< 0.01*

Note: *COPM* Canadian Occupational Performance Measure, *CFS* Cancer Fatigue Scale, *BDI* Beck Depression Inventory, The European Organization for Research and Treatment of Cancer Core Quality of Life Questionnaire C-30 and BR23 (EORTC QOL-C30 - EORT QOL-BR23); COPM and CFS averages (Paired student-t test), BDI and EORTC-QOL averages (Wilcoxon signed rank test); *p < 0.01

## Discussion

After identifying the activities of women with breast cancer that involve performance problems, following the OB-PSS training towards these activities carried out on women, it was observed that their activity performances and satisfactions as well as life qualities improved, but that their cancer-related fatigue and depression levels declined. In fact, as far as we know, this is the first study that shows the effects of OB-PSS on activity performance and satisfaction, fatigue, depression and quality of life in patients with breast cancer.

For a better understanding of functionality levels of survivors, it is important to determine what activities women with breast cancer mostly take part in during their daily routines and at what stage of these activities they encounter with problems. COPM is a scale that acts as a guide in person-based interventions that are to be carried out towards activity priorities and performance problems of survivors. In studies carried out into various cancer types, late middle-aged or elderly individuals mostly reported performance problems related to self care activities, while on the other hand middle-aged adults stated that they suffer from productivity problems beginning from the initial stages of treatment [32, 33]. In their studies aiming at the improvement of activity problems in women with breast cancer, Lyons et al. examined the strategies and discovered that patients mostly encounter with problems in paid or unpaid activities [8]. Considering the results of our study, participants of which are adult individuals with cancer at early stage, individuals were found to suffer from activity performance problems mostly in managing household activities (sweeping the floor, hanging curtains, cooking etc.). This shows great similarity to literature results and our study thus may serve to advancing the activity programs of clinicians and academicians working in the rehabilitation field.

PSS is a method that involves coping with problems or symptoms as well as difficulties faced in disease management, or making changes in life styles against them for better self-management. Various strategies are used in solving the problems that have been identified [34]. Lyons applied PSS to 80 activities in a study he conducted on 16 women with breast cancer as they were receiving chemotherapy, and stated that 40% of these activities worked well through activity adaptation as a way of solution [10]. In our study, it was seen that 58% of the participants chose an adaptive strategy of making changes to the implementation steps of the activity, which is supportive of similar studies in the literature as a consequence.

When studies into problems that lead to activity limitations in individuals with breast cancer are considered, due to the fact that certain side effects like fatigue, depression, nausea and shortness of breath, which influence functionality are seen in the early stages of the disease, it is said that the existence of movement restriction in the upper extremities as a result of the incomplete activity can cause problems in the activity performance [35, 36]. Considering the activities during which the participants experienced problems, it was seen that most of them needed the movement of upper extremities. Following the OB-PSS training, although movement limitation of individuals tended to prevail, it was established that their activity performance and satisfaction levels improved significantly thanks to the solution options developed through adaptive strategies. This improvement is highly important both for the patient and the therapist from the clinical point of view.

Fatigue and depression are the most frequent symptoms in cases of cancer. It has been suggested that problem solving strategies have been beneficial in improving the cognitive reconstructing strategy in males with local prostate cancer diagnosis and controlling the symptoms that are caused by cancer [37]. Another study on the physical and psychological effects that arose in 132 women with breast cancer also indicated that PSS were positively contributive to the process [38]. Our study has shown that OB-PSS has been effective in reducing the level of both depression and fatigue in women with breast cancer. We therefore recommend the use of OB-PSS within rehabilitation practices to be used against fatigue and depression that are commonly seen in survivors with breast cancer.

There are currently several studies into the evaluation of quality of life and effectiveness of intervention in cases of breast cancer [39–41]. One of these studies suggested that, after the application of chemical agents, almost all the women with breast cancer had impaired body perceptions, which affected their degree of overall life quality, and 20–25% of them had physical dysfunction and 30% of them suffered from sexual dysfunction [40]. As the continuity of daily routines is quite important, diversification of such practices in the rehabilitation process is necessary so as to positively influence the quality of life in women with breast cancer by making their lives more meaningful. OB-PSS training is an approach which backs up the formulation of solution options for the continuity of daily routines involving performance problems. Following the intervention process, we too have concluded that OB-PSS training has had positive influences on the parameters of quality of life as regards the individual’s functional and symptomatic conditions in both breast and other types of cancer.

As for the limitations involved in our study, firstly, the age distributions of the individuals included were not suitable for the examination of the treatment effectiveness by age. Secondly, although the number of samples is enough to show the statistical difference, we think it would be better to show the effectiveness of the intervention approach with the larger number of participants. Thirdly, behavioral changes have not been monitored through detailed observation in individual’s real-life environment (physical, social and cultural factors), which we regard as a negative point. In this context, it will make significant contributions to the interdisciplinary team with the expertise and experience in the oncological rehabilitation, with the OB-PSS training in which the detailed analysis of environmental factors is included in the larger number of survivors with breast cancer. Lastly, the lack of a control group can be said to be a limitation of our study. Therefore, we recommend that these limitations be considered in the planning of further studies with higher level of evidence.

## Conclusions

The activities that women with stage 1 and stage 2 breast cancer mostly experience performance problems are productivity, self-care and leisure time issues. Survivors who have performance problems in certain activities usually come up with solutions through developing adaptive strategies. With the improvement of these strategies, cancer-related fatigue and depression level in survivors has declined, while their activity performance, satisfaction and quality of life levels improved. Based on these results, it has been concluded that OB-PSS training could be used as an appropriate rehabilitation approach in women with breast cancer.

## Data Availability

Please contact the first author for data requests.

## Abbreviations

BDI: Beck Depression Inventory
CFS: Cancer Fatigue Scale
COPM: Canadian Occupational Performance Measure
EORT QOL-BR23: The European Organization for Research and Treatment of Cancer Core Quality of Life Questionnaire BR23
EORTC QOL-C30: The European Organization for Research and Treatment of Cancer Core Quality of Life Questionnaire C-30
OB-PSS: Occupation-based problem solving strategies
